# Preventive effect of probiotics supplementation on occurrence of gestational diabetes mellitus: A systematic review and meta-analysis of randomized controlled trials

**DOI:** 10.3389/fmed.2022.1031915

**Published:** 2022-12-01

**Authors:** Azin Pakmehr, Hanieh-Sadat Ejtahed, Nooshin Shirzad, Mahboobeh Hemmatabadi, Sara Farhat, Bagher Larijani

**Affiliations:** ^1^Endocrinology and Metabolism Research Center, Endocrinology and Metabolism Clinical Sciences Institute, Tehran University of Medical Sciences, Tehran, Iran; ^2^Obesity and Eating Habits Research Center, Endocrinology and Metabolism Clinical Sciences Institute, Tehran University of Medical Sciences, Tehran, Iran; ^3^Endocrine Research Center, Valiasr Hospital, Imam Khomeini Hospital Complex, Tehran University of Medical Sciences, Tehran, Iran; ^4^School of Medicine, Tehran University of Medical Sciences, Tehran, Iran; ^5^Students' Scientific Research Center, Tehran University of Medical Sciences, Tehran, Iran

**Keywords:** gestational diabetes mellitus, probiotics, gut microbiota, dysbiosis, prevention

## Abstract

**Background:**

Gestational diabetes mellitus (GDM) is a health challenge during pregnancy and is associated with adverse effects. Dysbiosis of the gut microbiota may play a role in developing inflammation and insulin resistance observed in GDM. Probiotics are supposed to be influential in preventing GDM since they can alter the composition of microbiota in the intestine. Despite the existing studies on the therapeutic effects of probiotics in women with GDM, in this study we aim to systematically review and meta-analyze the results of randomized control trials (RCTs) on the beneficial effects of probiotics supplements on the prevention of GDM in healthy pregnant women.

**Methods:**

Web of science, Scopus and PubMed databases were searched *via* a precise strategy to gather RCTs related to our study. Duplication removal, screening and data extraction were conducted by two researchers, independently. Quality assessment of eligible studies was conducted by Cochrane risk of bias tool. Meta-analysis was conducted using the random effects model due to substantial heterogeneity among studies.

**Results:**

Ten articles met our eligibility criteria from our initial search of 451 articles. Two thousand nine hundred and twenty-one participants without previously diagnosed glucose disturbance were included in our analysis. Probiotics reduced GDM incidence by 33% (RR = 0.67, 95% CI: 0.47, 0.95), while greater effect was detected in trials using multiple-strains probiotics (RR = 0.65, 95% CI: 0.42, 0.99). We did not detect any significant benefits or harms related to probiotics supplements on secondary outcomes including GDM related infantile and maternal complications including preeclampsia, caesarian section, mothers' weight gain during pregnancy, prematurity, macrosomia, hypoglycemia, NICU admission, and birth weight.

**Conclusion:**

Probiotics supplementation may reduce the incidence of GDM and help control glucose parameters in pregnant women. Further studies are warranted regarding the GDM-related maternal and infantile complications.

**Systematic review registration:**

https://www.crd.york.ac.uk/prospero/display_record.php?ID=CRD42022315550, identifier: CRD42022315550.

## Introduction

Gestational diabetes mellitus (GDM) is defined as abnormal glucose metabolism occurring during the second or third trimester of pregnancy and is one of the most common complications of the pregnancy (1). In Diabetes Atlas (2019), the International Diabetes Federation (IDF) estimates that 223 million women between 20 and 79 years suffer from diabetes. Besides, around 20 million or 16% of live births are affected by hyperglycemia during pregnancy (2). This challenge of maternal and child health increases the risk of preeclampsia, eclampsia, spontaneous abortion, macrosomia, shoulder dystocia, neonatal hyperglycemia and subsequent maternal metabolic syndrome (3).

Various risk factors have been diagnosed for GDM including ethnicity, advanced maternal age, increased body mass index (BMI), family history of type 2 diabetes mellitus (T2DM), and having a history of previous GDM (4). Moreover, Zhang and Ning accumulated data from multiple studies assessing maternal dietary intakes before and during pregnancy and found a great relationship between the dietary intakes and risk of GDM (5).

In a normal pregnancy, an inflammatory condition develops, helping adapt to the growing fetus, which alters insulin receptor signaling and results in an increased insulin resistance (6). On the other hand, higher amounts of interleukin-6 (IL-6) and tumor necrosis factor-α (TNF-α) and lower maternal level of adiponectin were shown to be associated with GDM (7–9). Evidence regarding the composition of gut microbiota in pregnant women indicated a great change along with adiposity, inflammation and insulin resistance (10, 11). Koren et al. reported that the gut dysbiosis observed in late pregnancy resembles the gut microbiota composition in metabolic disorders (12). Also, changes in the gut microbiome in pregnant women with GDM go beyond a normal pregnancy and gut microbiota in GDM may be similar to non-pregnant women with T2DM (13, 14). According to the contribution of gut dysbiosis in developing metabolic disorders, probiotics were used to maintain the balance of the composition of gut microbiota (15). Probiotics are defined as live microorganisms benefiting host's health when administered in sufficient amounts (16). Hu et al. in a meta-analysis conducted on 12 randomized control trials (RCTs) indicated that probiotics could significantly lower the glucose level in diabetic patients (17). Since changes in insulin sensitivity and microbiota composition seem to be similar in GDM and T2DM, probiotics supplementation has been suggested as an intervention to prevent and control GDM.

Despite the existing systematic reviews and meta-analyses in order to assess the therapeutic effects of probiotics on pregnant women with GDM (18, 19), there is a few systematic reviews and meta-analyses to determine whether probiotics could prevent the incidence of GDM in healthy pregnant women (20–22). Moreover, there is a need for evaluating the effect of probiotics supplementation on maternal and fetal consequences of GDM. So, we conducted an updated systematic review and meta-analysis to comprehensively review all relevant RCTs assessing the effect of probiotics on the prevention of GDM and related complications during pregnancy.

## Materials and methods

We aimed to evaluate the efficacy of probiotics supplementation on GDM prevention and its maternal and infantile impacts among pregnant women with pre-pregnancy normal glucose level. In order to report the findings, Preferred Reporting Items for the Systematic reviews and Meta-analysis for Protocol (PRISMA-P) was followed (23). This systematic review and meta-analysis protocol was registered in PROSPERO with ID: CRD42022315550 in March 2022.

### Search strategy

A comprehensive search was conducted to identify the relevant literatures using Web of science, Scopus and PubMed databases up to September 2022. The following keywords and their combinations were applied to develop a systematic search strategy through the databases: (Probiotic[Mesh] OR Probiotics[Mesh] OR Probiotic[tiab] OR Probiotics[tiab] OR synbiotics[tiab] OR lactobacillus[tiab] OR lactobacilli[tiab] OR bifidobacteria[tiab] OR bifidobacterium[tiab]) AND (“Diabetes, Gestational”[Mesh] OR “gestational diabetes mellitus”[tiab] OR “gestational diabetes”[tiab] OR “diabetes, pregnancy-induced”[tiab] OR “pregnancy-induced diabetes”[tiab] OR “pregnancy induced diabetes”[tiab] OR GDM[tiab] OR “diabetes mellitus gestational”[tiab]). The references of relevant review studies were searched manually. The language was not considered as a restriction.

### Eligibility criteria and study selection

Screening started after the removal of automatic and manual duplicates. Afterwards, the titles and abstracts of the retrieved records were screened to select potentially eligible studies. The researchers went through the full-text of the remained papers to confirm the relevance.

Finally, RCTs that allocated pregnant women without GDM to an intervention group receiving probiotics supplements or to a control group receiving placebo and reported at least one of the following outcomes were included: (1) Occurrence of GDM and blood glucose indicators (as the primary outcomes of this systematic review), (2) GDM related infantile and maternal complications including preeclampsia, caesarian section, mothers' weight gain during pregnancy, prematurity, macrosomia, hypoglycemia, NICU admission, and birth weight (as the secondary outcomes of this systematic review). Probiotics could be administered alone or in combination with prebiotics which is referred to as synbiotics.

The PICO of this meta-analysis is as follows:

Population: Women with normal glucose levels prior to pregnancy

Intervention: Probiotics foods and supplements

Comparator: Placebo or no probiotics used

Outcome: Occurrence of GDM or other adverse outcomes as secondary outcomes

Type of study: Clinical trials

Studies were excluded if the women were diagnosed with glucose imbalance before pregnancy. Observational studies, editorial, letters, reviews or systematic reviews, and animal studies were also excluded. Study selection was conducted by two independent researchers. Disagreements between the two investigators were resolved by discussing until reaching consensus.

### Data extraction and quality assessment

Extraction of data which comprised first author of the trial, year of publication, type of study, country, method of diagnosing GDM, size of the groups, population characteristics, intervention details and outcome measurements was done by two researchers independently and any disagreements were solved through consensus.

The outcomes of this meta-analysis comprise GDM incidence as a primary outcome and other maternal factors including fast blood sugar (FBS), 1 and 2 h blood glucose after GDM test, preeclampsia, caesarian section and weight gain during pregnancy. Infantile outcomes including prematurity, macrosomia, hypoglycemia, NICU admission and birth weight were evaluated as well.

The methodological quality of each clinical trial was assessed using Cochrane risk of bias tool (24). Random sequence generation, allocation concealment, blinding of participants/personnel, blinding outcome assessment, incomplete outcome data, and selective outcome reporting were six domains that have been considered in this tool.

We also used the Grading of Recommendations, Assessment, Development, and Evaluation (GRADE) framework to assess the certainty of evidence for each assessed outcome.

### Statistical analysis

Risk Ratio (RR) and 95% confidence interval were calculated for binary outcomes, i.e., GDM, caesarian section, hypoglycemia, macrosomia, preeclampsia, prematurity, NICU. The DerSimonian–Laird random effects model was utilized for the meta-analysis. When at least 10 papers were available, publication bias was evaluated using a visual inspection of the funnel plot and Egger's regression test (25). Substantial heterogeneity was established according to an *I*^2^ ≥ 50 and a *P*-value cutoff of 0.10 for Cochran's *Q*-test. To investigate the robustness of the pooled effect sizes, we performed influence analysis by excluding each cohort one at a time. Moreover, subgroup analyses have been performed according to the baseline BMI of mothers as well as single- or multi-strain probiotics used for supplementation. All analyses were carried out using Stata 15 (Stata Corp. College Station, Texas, USA). Results were considered statistically significant if *P*-value < 0.05.

## Results

**Figure 1:**
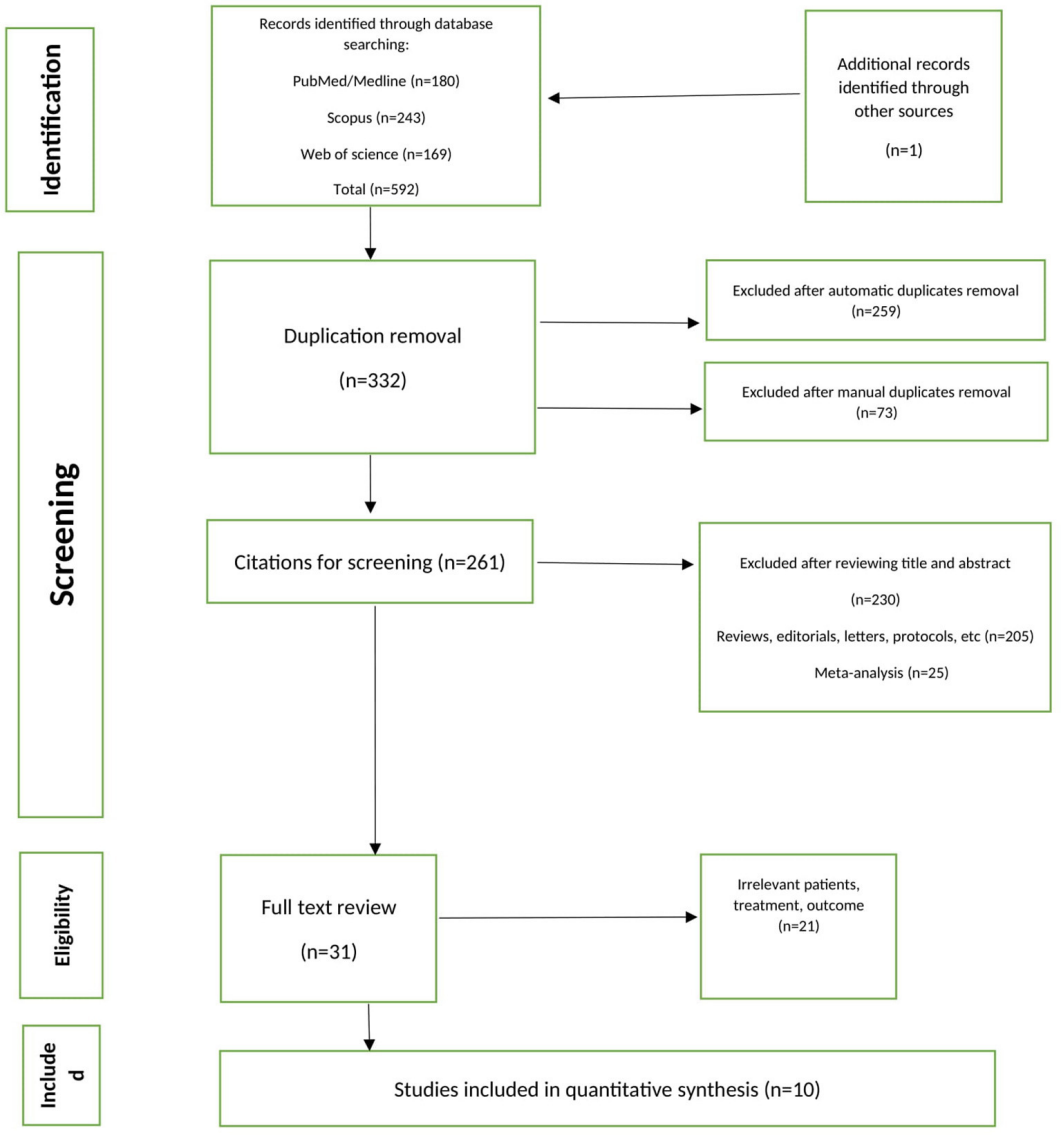
Flowchart of the studies selection.

Our initial search yielded a total of 592 articles (180 from MEDLINE/PubMed, 169 from Web of Science, 243 from Scopus, and 1 from manual searching). After automatic and manual duplicate elimination, 261 articles were retained. After eliminating 230 publications using title and abstract screening, we evaluated the full-text of the remaining research. As illustrated in Figure 1, 10 RCTs were included in this meta-analysis.

### Characteristics of the included studies

Table 1 indicates the characteristics of the included studies. All studies were parallel and blinded RCTs. Eight studies had two arms including intervention and placebo arms. One study had an additional arm for the control group (27). One study had four arms for two interventions (21).

**Table 1:** Characteristics of the included studies.

**References**	**Study design**	**Country Diagnosis of GDM**	**Sample size (Intervention /Placebo)**	**Population characteristics**		**Intervention**		**Outcomes**	**GDM occurrence (%) (Intervention/ Placebo)**
				**Gravida status**	**Age**	**Probiotics species**	**Vehicle Dosage Frequency Duration**		
Wickens et al. (20)	Double-blind, Randomized placebo-controlled parallel trial	Newzealand 2 h-75g-OGTT	194/200	Pregnant woman with a personal or partner history of atopic disease 14–16 weeks	34/34 IQR: (30–36/31–37)	I: *L. rhamnosus* (HN001) P: maize-derived maltrodextrin	- Capsule - 6 × 10^9^ CFU - Once daily - 8–16 weeks	- FBS↓ - Birth weight↔ - Macrosomia↔ - Prematurity↔ - NICU↔ - CS↔	2.1/6.5 ↓
Asgharian et al. (26)	Triple-blind, randomized placebo-controlled two-parallel trial	Iran 2 h-75 g-OGTT	64/64	Pregnant women BMI ≥ 25 kg/m^2^ Age ≥ 18 24 weeks	29.5 ± 6.2/29.4 ± 5.5	I: yogurt with additional *Lactobacillus acidophilus* and *Bifidobacterium lactis* P: yogurt	- Yogurt - 100 g/day - 5 × 10^10^ CFU - Daily - 4 weeks	- FBS↓ - Birth weight↔ - Macrosomia↔ - Prematurity↔ - NICU↔ - Preeclampsia↔ - CS↔ - Weight gain↔	9/17 ↔
Ebrahimzade et al. (27)	Double-blind, randomized Placebo-controlled two-parallel trial	Iran 2 h-75 g-OGTT	80/82	Pregnant women, singleton aged ≥ 16 no metabolic disorders 14–16 weeks	30 ± 6.7	I: *lactobacilus bifidiom* and *streptococcus* P: corn starch	- Capsule - 500 mg - Once daily - 12 weeks	FBS↓	26.3/ 59.8/ 67.1(C) ↓
Pellonperä et al. (21)	Double-blind, placebo-controlled randomized trial	Finland 2 h-75g-OGTT	99/91	Pregnant women BMI ≥ 25 kg/m^2^ Abscent of chronic diseases 13.9 ± 2.1 weeks	30.8 ± 4.8/ 30.4 ± 4.1	I: *Lactobacillus rhamnsus* and *bifidobacterium* P: micro crystallin cellulose	- Capsule - 2.4 g/10^10^CFU - Once daily - 12.5 ± 3.1 - Weeks	- FBS↓ - Birth weight↔ - Macrosomia↔ - Prematurity↔ - NICU↔ - Preeclampsia↔ - CS↔ - Hypoglycemia↔	35.4/39.6 ↔
Halkjær et al. (22)	Randomized double-blind controlled study	Denmark 2 h-75 g-OGTT	25/24	Nulliparous singleton pregnant women with 30 ≤ BMI < 35 kg/m^2^ 14–20 weeks	30.7 ± 4.5/30.7 ± 4.7	I: *streptococus thermophilus, Bifidobacteria, Lactobacilli* P: microcrystalline cellulose, magnesium stearate, silicon dioxide	- Capsule - 45 × 10^10^ CFU - Two twice - 7–16 weeks	- FBS↔ - Birth weight↔ - Macrosomia? - Prematurity? - Preeclampsia? - CS? - Weight gain↔	16/8 ↔
Callaway et al. (28)	Double-blind randomized controlled Trial	Australia 2 h-75 g-OGTT	207/204	Singleton pregnant women, BMI ≥ 25 kg/m^2^ 15.9 weeks	31.3 ± 4.7/31.7 ± 4.8	I: *Lactobacillus rhamnosus* and *Bifidobacterium animalis* P: microcrystallin cellulose and dextrose anydrate	- Capsule - >10^9^ CFU - Once daily - 12 weeks	- FBS↓ - Birth weight↔ - Macrosomia↔ - Prematurity↔ - Preeclampsia↔ - CS↔ - Weight gain↔ - Hypoglycemia↔	12.3/18.8 ↔
Lindsay et al. (29)	Double-blind, placebo-controlledrandomized trial	Ireland 3 h-100-OGTT	62/74	Singleton pregnant women 30 < BMI < 39.9 kg/m^2^ 24 weeks	31.4 ± 5/31 ± 5.2	I: *Lactobacillus salivarius*	- Capsule - 100 mg of 10^9^CFU - Once daily - 28 weeks	- FBS↔ - Birth weight↔ - Macrosomia↔ - Prematurity↔ - NICU↔ - Preeclampsia↔ - CS↔ - Weight gain↔	16.1/14.9 ↔
Godfrey et al. (30)	Double-blind randomized controlled trial	Singapore, NewZealand,UK 2 h-75 g-OGTT	294/283	Women planning to conceive within 6 months	30.53 ± 3.40/30.14 ± 3.30	I: *Lactobacillus rhamnosus* and *Bifidobacterium animalis* P: Folic acid, iron, calcium, iodin, beta caroten	- Suchet - 10^10^ CFU - Twice daily - 52 weeks	- FBS↔ - Birth weight↔ - Macrosomia↔ - Prematurity↓ - NICU↔ - CS↔ - Hypoglycemia↔	22.6/24.8 ↔
Shahriari et al. (31)	Parallel double-blind, randomized, placebo-controlled clinical trial	Iran 2 h-75 g-OGTT	241/266	Singleton pregnancy 18.5 ≤ BMI ≤ 39.9 kg/m^2^ 14 weeks	31.83 ± 5.80/32.20 ± 5.51	I: *Lactobacillus acidophilic, Bifidobacterium longum* and *Bifidobacterium bifidum* P: starch and maltodextrins	- Capsule - 7.5/1.5/6 × 10^9^ CFU - Once daily - 14–24 weeks	- -FBS↔ - Birth weight↔ - Macrosomia↔ - Preeclampsia↔ - -CS↔	41.9/40.2 ↔
Louto (32)	Double-blind, placebo-controlled clinical trial	Finland 2 h-75 g-OGTT	85/86	No chronic metabolic diseases; except allergic diseases first trimester	29.7 ± 4.1/30.1 ± 5.2	I: *Lactobacillus rhamnosus* GG and *Bifidobacterium* *lactis* Bb12 p: microcrystalline cellulose and dextrose anhydrate	- Capsule - 10^10^CFU - Once daily - 40 weeks	- Preterm↔ - CS↔ - Birth weight↔	13/36 ↓

I, intervention; P, placebo; OGTT, oral glucose tolerance test; CFU, colony forming unit; FBS, fast blood sugar; GDM, gestational diabetes mellitus; NICU, neonatal intensive care unit; CS, caesarian section.

The number of participants who finished each study ranged from 49 to 507. Totally, 2,921 pregnant women from New Zealand, Iran, Finland, Denmark, Australia, Ireland, Singapore, and the United Kingdom were recruited. All studies included pregnant women without diagnosed diabetes at the beginning. Seven studies included only singleton pregnancy, one study started 6 months pre-pregnancy (30) and two studies had no limitations for multiple pregnancies (20, 32). One study included women with their or partner's history of atopic disease (20). Three studies included only overweight and obese participants (21, 26, 28), two studies evaluated only obese women (22, 29), although five others didn't exclude women based on their BMI.

All the studies compared probiotics vs. placebo. One study included a fish oil capsule (21) and one study included dietary intervention as well as probiotics (32). The intervention types are probiotics capsules in 8 studies, probiotic yogurt in one study (26) and probiotics sachet in another one (30). The frequency of intervention in most studies was once daily except for two studies which were twice a day (22, 30). The dose of probiotics used in the studies varies. Mostly were more than 10^9^ CFU/d and for two studies the exact dose was not declared (27, 30). Eight studies used multiple species and two studies used only a single species probiotic (20, 29). The species were *Lactobacillus rhamnosus, Lactobacillus acidophilus, Lactobacillus salivarius, Bifidobacterium lactis, Bifidobacterium longum, Bifidobacterium bifidum, Lactobacillus casei, Lactobacillus bulgaricus, Lactobacillus plantarum, Lactobacillus paracasei, Bifidobacterium breve, Bifidobacterium infantis*, and *Streptococcus thermophilus*. In one study (30), the intervention began within 6 months prior to pregnancy, in seven studies before 20 weeks, and in two studies beyond 20 weeks (26, 29).

All of the studies reported the incidence of GDM as the primary outcome mostly on the basis of 2 h-75 g-OGTT except for one study using 3 h-100 g-OGTT (29). Nine studies reported data on birth weight (20–22, 26, 28–32), eight studies recorded macrosomia (20–22, 26, 28–31), seven studies reported prematurity (20–22, 26, 28, 30, 32), five studies reported NICU admission (20, 21, 26, 28, 29), three studies reported hypoglycemia (21, 28, 30), three studies reported weight gain (22, 26, 29), nine studies reported caesarian section (20–22, 26, 28–32) and six studies reported preeclampsia as the secondary outcomes (21, 22, 26, 28, 29, 31).

### Findings from systematic review

Ten articles were included in our systematic review, nine of which reported data on FBS (20–22, 26–31). The overall trend appears to be downward; nevertheless, five investigations demonstrated a significant decrease in fasting blood glucose (20, 21, 26–28). The study conducted by Callaway et al. revealed a difference of 0.1 mmol/l in FBS prepost alterations between the probiotics supplement group and the placebo group (28). In a research done by Ebrahimzade et al. FBS dropped by 0.3 mmol/l more in the probiotics group than in the control group (27). Reports on 2-h-OGTT presented in eight studies (20–22, 26–28, 30, 31) and four studies reported a slight decrease following probiotics although they were not significant (20, 21, 26, 27).

Nine papers published data on birth weight (20–22, 26, 28–32). Seven of them reported slightly larger infants in probiotic groups ranging from 10 g in the paper by Asgharian et al. to 112 g in the paper by Louto et al., although these differences were not statistically significant (26, 32). Three researches looked on maternal weight gain during pregnancy (22, 26, 29). In two studies, women in the probiotics group gained more weight than those in the placebo group, despite the differences not being statistically significant (26, 29).

### Findings from meta-analysis

**Figure 2:**
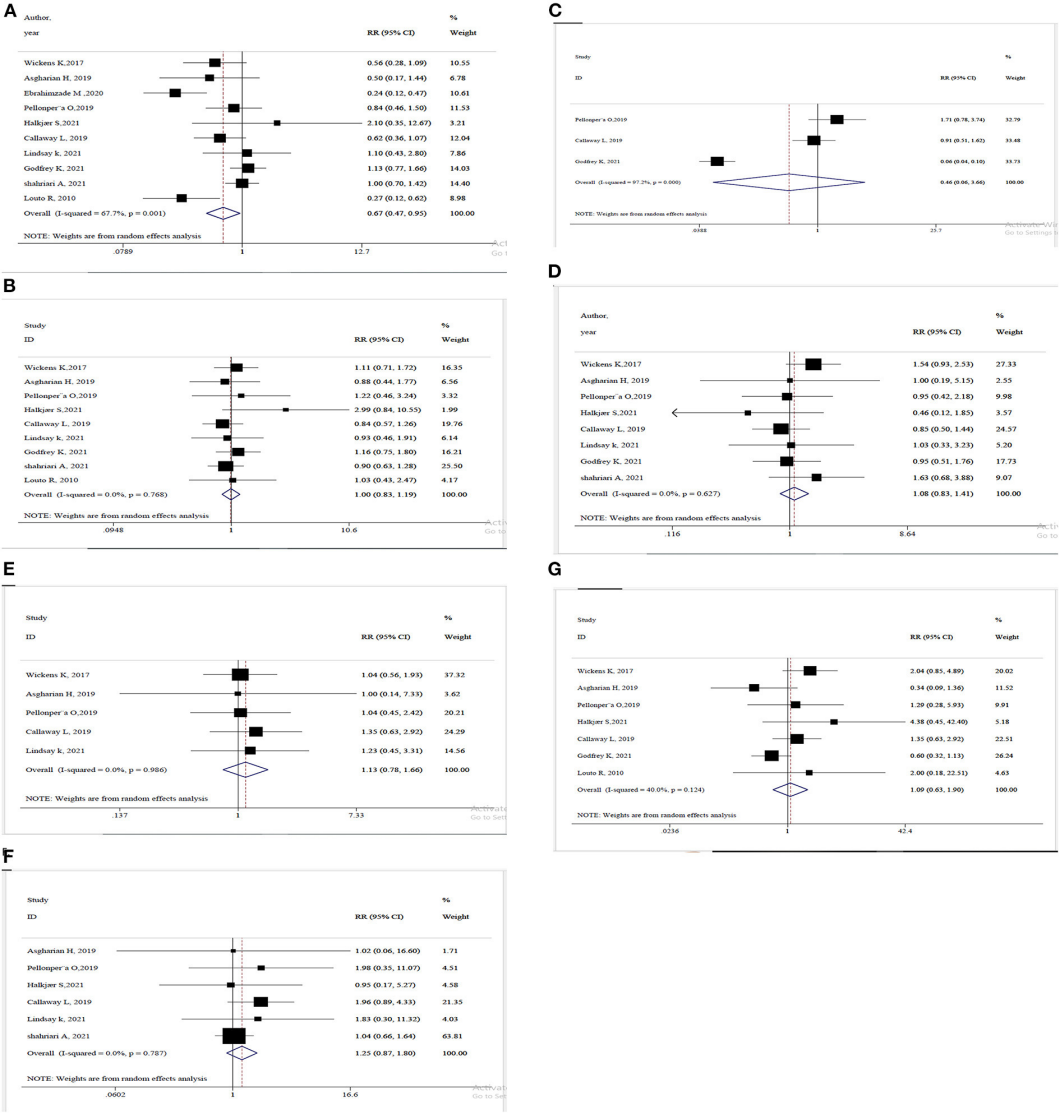
Forest plot more the meta-analysis of **(A)** GDM/ **(B)** Cesarean section/ **(C)** Hypoglycemia/ **(D)** Macrosomia/ **(E)** NICU admission/ **(F)** Preeclampsia/ **(G)** prematurity.

The outcomes of the meta-analysis are depicted in Figure 2. All 10 investigations on the incidence of GDM revealed that probiotics supplements lowered the risk by 33% significantly (RR = 0.67, 95% CI: 0.47, 0.95). Heterogeneity was statistically significant regarding the GDM (*I*^2^ = 67.7%, *p* < 0.001), and sensitivity analyses have been shown that pooled RR is dependent on the results of Wickens et al. (20), Ebrahimzade et al. (27), Luoto et al. (32), and Callaway et al. (28) studies. Excluding the result of Asgharian et al. (26) from analysis, the only study which prescribed probiotic yogurt for 4 weeks instead of a probiotic supplement, did not change the significance of the result of meta-analysis (RR = 0.68, 95% CI: 0.46, 0.99). Regarding the duration and time of onset of the intervention, in Godfrey et al. (30) study, probiotics, as well as myo-inositol and multiple micronutrients were taken during preconception and throughout the pregnancy and this study has the longest period of intervention. The result of the meta-analysis was also robust by omitting this study (RR = 0.61, 95% CI: 0.41, 0.90) (Supplementary material).

Sub-group analyses were conducted according to the baseline BMI of mothers as well as single- or multi-strain probiotics used for supplementation (Table 2). A slight greater effect on GDM occurrence was detected in eight trials using multi-strain probiotics (RR = 0.65, 95% CI: 0.42, 0.99), however, the heterogeneity was still existed within this sub-group (*I*^2^ = 73.5%, *p* < 0.001).

**Table 2:** Results of subgroup-analysis based on mothers' weight status before pregnancy and number of probiotic strains.

		**Meta-analysis** ^*^				**Heterogeneity**		
**Study group**	**Number of studies**	**RR (95% CI)**			***P*** **-effect**	***I^2^*** **(%)**	***P*** **-within group**	***P*** **-between group**
**GDM**								
**Mothers weight**								0.40
Normal weight	5	0.56	0.31	1.02	0.06	83.9	<0.001	
Overweight or obese	5	0.76	0.54	1.06	0.11	0.0	0.54	
**Probiotic strains**								0.77
Single strain	2	0.72	0.38	1.39	0.33	26.6	0.24	
Multiple strains	8	0.65	0.42	0.99	0.04	73.5	<0.001	
**Macrosomia**								
**Mothers weight**						0.10
Normal weight	3	1.32	0.93	1.89	0.12	0.0	0.43	
Overweight or obese	5	0.86	0.58	1.26	0.43	0.0	0.91	
**Probiotic strains**						0.14
Single strain	2	1.44	0.91	2.28	0.12	0.0	0.52	
Multiple strains	6	0.95	0.69	1.30	0.73	0.0	0.74	
**Prematurity**								
**Mothers weight**						0.95
Normal weight	3	1.15	0.43	3.08	0.78	62.5	0.07	
Overweight or obese	4	1.10	0.49	2.48	0.81	33.0	0.21	
**Probiotic strains**						0.13
Single strain	1	2.04	0.85	4.88	0.11	-	-	
Multiple strains	6	0.92	0.52	1.62	0.76	28.3	0.22	
**NICU**								
**Mothers weight**						0.72
Normal weight	1	1.04	0.56	1.93	0.90	-	-	
Overweight or obese	4	1.19	0.74	1.93	0.46	0.0	0.97	
**Probiotic strains**						0.82
Single strain	2	1.09	0.64	1.84	0.75	0.0	0.78	
Multiple strains	3	1.19	0.69	2.05	0.54	0.0	0.89	
**Preeclampsia**								
**Mothers weight**						0.19
Normal weight	1	1.04	0.66	1.64	0.87	-	-	
Overweight or obese	5	1.72	0.94	3.16	0.08	0.0	0.94	
**Probiotic strains**						0.67
Single strain	1	1.83	0.29	11.32	0.51	-	-	
Multiple strains	5	1.23	0.84	1.78	0.8	0.0	0.78	
**CS**								
**Mothers weight**								0.70
Normal weight	4	1.02	0.82	1.28	0.83	0.0	0.81	
Overweight or obese	5	0.95	0.71	1.27	0.75	0.0	0.76	
**Probiotic strains**						0.72
Single strain	2	1.06	0.73	1.54	0.75	0.0	0.68	
Multiple strains	7	0.98	0.80	1.20	0.84	0.0	0.59	

RR, risk ratio; GDM, gestational diabetes mellitus; NICU, neonatal intensive care unit; CS, caesarian section.

* All analyses were conducted using random-effects model.

Nine studies reported data on incidence of caesarian section but no association were found according to statistics (RR = 1.00, %95CI: 0.83, 1.19). In terms of macrosomia, 8 studies evaluated the effects of this intervention and found no statistically significant association (RR = 1.08, %95CI: 0.83, 1.41). Three studies reported data regarding the effects of probiotics on hypoglycemia incidence and the result was not significant (RR = 0.46, 95%CI: 0.06, 3.66). Five studies evaluated whether probiotics supplements affect NICU admission and according to meta-analysis, there was no statistically significant relationship (RR = 1.13, 95%CI: 0.78, 1.66). We found no significant association between probiotics and preeclampsia analyzing the results of 6 studies (RR = 1.25, 95%CI: 0.87, 1.80). Also, combining the results of 7 studies, no statistically significant association was found between probiotics and the incidence of prematurity (RR = 1.09, 95%CI: 0.63, 1.90). In addition, sensitivity analyses were performed for all other outcomes in meta-analyses, demonstrating that the results were robust. Moreover, in order to remove between-study heterogeneity, sub-group analyses were performed based on the baseline BMI of mothers as well as single- or multi-strain probiotics and no significant association was found regarding the GDM-related complications (Table 2).

### Quality assessment of the studies

**Figure 3:**
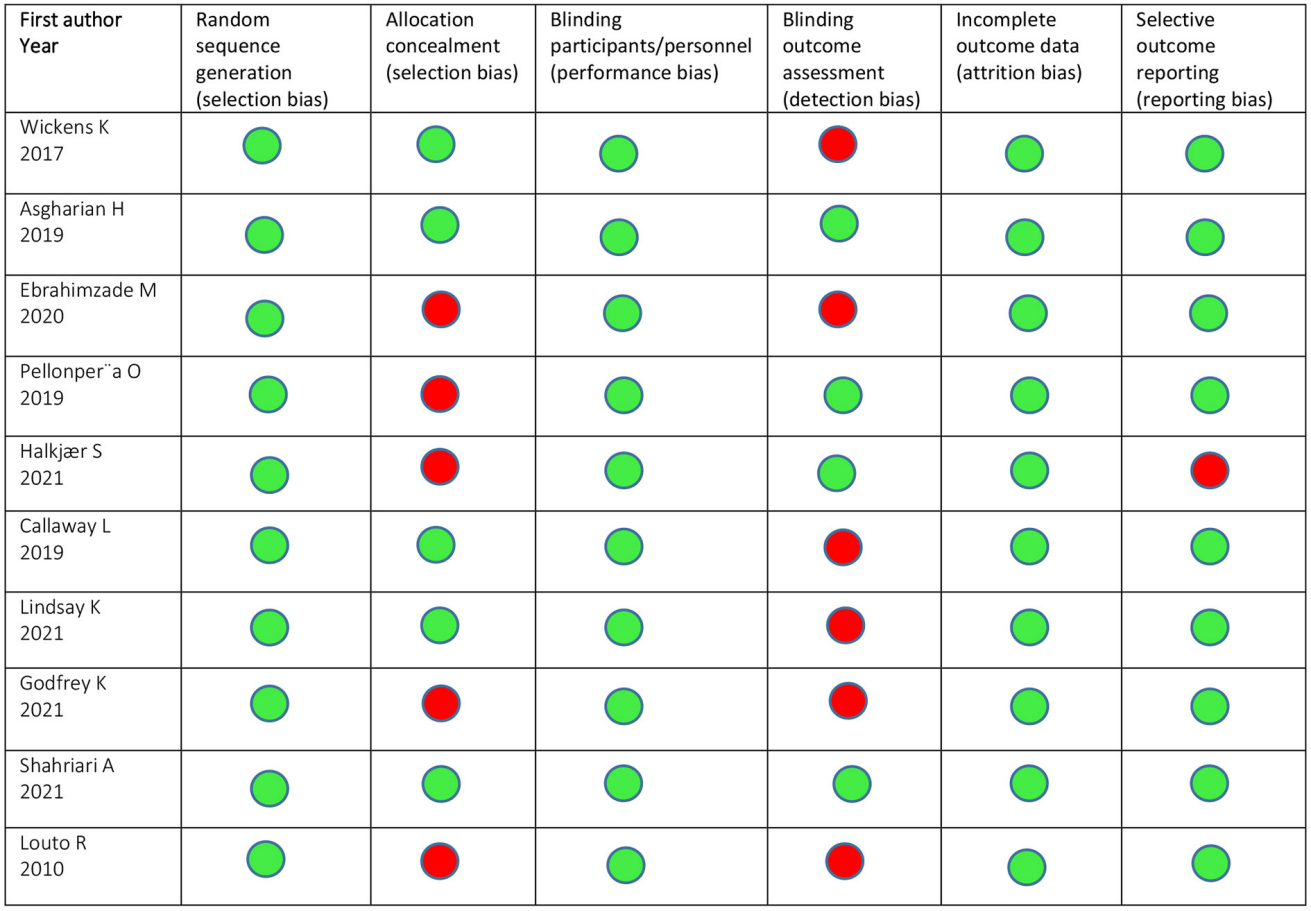
Quality assessment of the included studies.

The methodological quality and risk of bias of each study is shown in Figure 3. Selection bias, performance bias and attrition bias were not reported in any of the included studies. Allocation concealment was conducted in half of the studies (20, 26, 28, 29, 31) and in four studies the outcome assessors were blinded (21, 22, 26, 31). Moreover, one study was evaluated as high risk for selective outcome reporting (22). Overall risk-of-bias for two studies was low in all domains (26, 31). Four studies were judged to have a high risk of bias in one domain, 3 in blinding outcome assessors (20, 28, 29) and one in allocation concealment (21). Three trials were judged to have some concerns for both blinding outcome assessors and allocation concealment (27, 30, 32). One study had a high risk in allocation concealment and selective outcome report (22).

### Publication bias and certainty of the evidence

**Figure 4:**
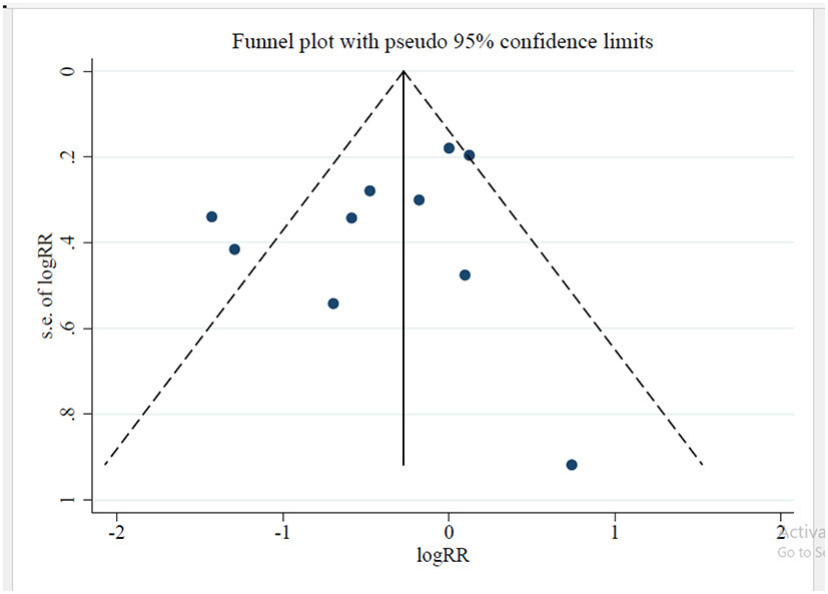
Funnel plot for GDM showing no publication bias.

Because the number of studies with other outcomes was <10, the funnel plot was drawn only for GDM which there was no indication of publication bias (Figure 4).

According to the GRADE assessment, the certainty of the evidence was rated as very low to moderate (Table 3). The quality of the body of evidence regarding GDM and NICU occurrence is low due to existed risk of bias and inconsistency of the results of included trials. It means that the likelihood that the effect might be substantially different is considerable. Regarding the preeclampsia, caesarian section, prematurity, and macrosomia, the certainty of the evidence was moderate and the true effect is probably close to the estimated effect. The quality of evidence was rated very low just for hypoglycemia because of the low number of included studies in this regard.

**Table 3:** Quality of evidence using GRADE assessment.

**Outcome**	**No. of studies**	**Risk of bias**	**Inconsistency**	**Indirectness**	**Imprecision**	**Publication bias**	**Quality**	**No of participants**		**Effect**
								**Intervention**	**Control**	**Pooled effect size RR (95%CI)**
GDM	10	Serious	Serious	Not serious	Not serious	Not serious	⊕⊕○○ Low	1,329	1,356	0.66 (0.46, 0.95)
Macrosomia	8	Serious	Not serious	Not serious	Not serious	Undetected	⊕⊕⊕○ Moderate	1,192	1,217	0.08 (0.83, 1.41)
Prematurity	7	Serious	Not serious	Not serious	Not serious	Undetected	⊕⊕⊕○ Moderate	956	932	1.09 (0.63, 1.90)
NICU	5	Serious	Not serious	Not serious	Serious	Undetected	⊕⊕○○ Low	618	611	1.13 (0.78, 1.66)
Preeclampsia	6	Serious	Not serious	Not serious	Not serious	Undetected	⊕⊕⊕○ Moderate	694	724	1.25 (0.87, 1.80)
CS	9	Serious	Not serious	Not serious	Not serious	Undetected	⊕⊕⊕○ Moderate	1,269	1,294	0.99 (0.83, 1.19)
Hypoglycemia	3	Serious	Serious	Not serious	Serious	Undetected	⊕○○○ Very low	590	581	0.46 (0.06, 3.66)

RR, risk ratio; GDM, gestational diabetes mellitus; NICU, neonatal intensive care unit; CS, caesarian section.

## Discussion

GDM as a crucial health problem has been a challenge in recent years and efforts toward preventing and managing this threat are still continuing (33). Our systematic review and meta-analysis included 10 studies evaluating the effects of probiotics supplements on the incidence of GDM, glycemic parameters and some maternal and infantile complications in pregnant women without GDM. Our meta-analysis revealed that the probiotics supplementation decreases the incidence of GDM by 33%, showing the preventive role of probiotics on GDM. This effect was 35% for multi-strain probiotics. In 2019, Chatzakis et al. collected data from 23 RCTs evaluating the effects of several interventions on preventing GDM -including four studies regarding probiotics- and found nothing significantly effective (34). On the other hand, there is another study in 2019 which indicated a significant reduction in the risk of GDM following probiotics supplementation during early pregnancy (35). These contradictory findings could be due to different eligibility criteria for selecting the articles and the heterogeneities in quality and methodology of the included studies such as differences in ethnicities, baseline characteristics and past medical history of participants, various probiotics dosages and bacterial species, different modes of delivery, duration and frequency of probiotics administration.

Regarding the various effects of different probiotics species used, the study in New Zealand showed a significant effect of *L. rhomnosus* on GDM incidence but *L. salivarius* had no significant effect on this risk in Ireland (20, 29). Notably, all of the included studies in our meta-analysis with significant decrease in the GDM incidence started supplementation before 20 weeks of pregnancy (20, 27, 32) while the studies by Asgharian et al. and Lindsay et al. which started after 20 weeks of pregnancy found no significant effect on the incidence of GDM (26, 29). Furthermore, the dosage of intervention may influence the effect as 10^9^ CFU/day or higher was suggested to be more efficient in lowering glucose markers (36) although all the studies included in our meta-analysis used more than 10^9^ CFU. All three studies that found significant decline following probiotics used capsules for administration which is more feasible to achieve desirable and accurate dose (20, 27, 32). On the other hand, yogurt and suchet seemed to be more patient-dependant and none of the two studies utilizing them found any difference (26, 30). Also, the frequency of intervention was twice a day for two studies which did not show any significant difference in results comparing to others and more studies are required to determine whether the frequency affects the result of intervention (22, 30). Besides, in the studies done by Wickens et al. and Ebrahimzade et al., which seem to have effective results on GDM incidence according to sensitivity analysis, FBS decreased significantly as was shown in a recent meta-analysis by Łagowska et al. on pregnant women with GDM (37). Sub-group analysis has been shown that multi-strain probiotics was slightly more effective on GDM occurrence and the effect of baseline BMI of mothers was not significant. Sensitivity analyses have been shown that result of the meta-analysis was robust after omitting the studies which prescribed probiotics food as an intervention or started supplementation in women planning to conceive within 6 months.

Despite several studies investigating the impact of probiotics on metabolic factors, the accurate mechanism is still unclear (38). Modulating gut microbiota composition as a result of using probiotics may be a key underlying mechanism (15). Fuller et al. demonstrated a positive relationship between glucose homeostasis during pregnancy and concentration of short chain fatty acids (SCFAs) as the main product of gut microbiota fermentation (39). SCFAs were shown to improve insulin sensitivity and correlate positively with Glucagon like peptide 1 (GLP-1) (40). Multiple studies found a decrease in production of SCFAs in T2DM resulting from lacking bacterial species known to produce these metabolites (41, 42). Similar alteration occurs in the gut microbiota of pregnant women with GDM leading to a lower amount of SCFAs which suggests a promising future for probiotics to play a role in preventing or treating this disease (43). Moreover, many studies have investigated the role of the inflammatory system in insulin resistance (44). In 2009, Wellen and Hotamisligil explained several ways through which inflammatory cytokines including TNFα interferes with insulin signaling pathways (45). As it was shown in multiple documents, probiotics could decrease the level of inflammatory markers and subsequently increase insulin sensitivity *via* improving the gut barrier functions and decreasing the translocation of bacterial lipopolysaccharides (46, 47). Our study couldn't find significant changes in the other outcomes following probiotics supplements which may be related to the different design of the studies. In the study by Godfrey et al. there was a significant decrease in prematurity following probiotics supplements which opposes the results found in a recent study by Jarde et al. (16). This discrepancy could be attributed to the duration of intervention which started about 6 months before pregnancy. Also, urogenital infection has been confirmed to contribute to preterm birth, and probiotics especially some *Lactobacillus* species which were used in this study seems to reduce the risk of this infection (48). Although the result of our meta-analysis could not show significant changes in prematurity following probiotics supplementation, further studies are needed to determine the exact role of probiotics in preventing preterm birth. Furthermore, our meta-analysis did not show any significant differences regarding the effects of probiotics on the incidence of macrosomia, hypoglycemia and NICU admission in comparison with placebo group. Although studies evaluating pregnant women with GDM represented the same results (37), however, considering that our results is mainly based on low-level evidence from limited number of clinical trials, more investigations on different types of probiotics are needed. Ilmonen et al., in a RCT demonstrated that dietary counseling along with probiotics from early pregnancy have beneficial effects on central adiposity and waist circumference in pregnant women but did not alter gestational weight gain significantly (49) which is in line with the results of our meta-analysis.

Moreover, none of our included studies show a difference between the side effects of the intervention group and placebo group. Gastrointestinal symptoms were the most common adverse effects relating to capsule intake. Evidence has been showing adverse association between moderate and high intake of probiotics and preeclampsia. Specifically, *L. rhamnosus* seems to modify inflammatory responses involved in developing preeclampsia (50, 51). However, our meta-analysis did not show significant reduction in preeclampsia as a result of probiotics supplementation and more studies are required to determine whether the dose or type of intervention contribute to this result.

A meta-analysis on eight clinical trials investigating the effects of specific type of probiotics on the incidence of caesarian section in pregnant women indicated no significant changes (52). This result is in line with our study which measured the effects of various mixtures of probiotics species.

The included studies that had high risk of bias in one or two domains were in line with studies by Asgharian et al. and Shahriari et al. which were judged to be of high quality in our meta-analysis and showed significant decrease in FBS along with small decline in GDM incidence (26, 31).

The main strength of this systematic review and meta-analysis is the comprehensive search and the relatively higher number of studies included in comparison with previous meta-analyses. Furthermore, through consideration of strict inclusion criteria, selective data about the effects of probiotics on healthy pregnant women was obtained. Taking this into account, we avoided possible biases which may develop by the presence of previous glucose disturbances. However, the results should be interpreted with considering the following limitations. First, we were not able to investigate publication bias for most of our outcomes except GDM due to the small number of studies. Second, causes for the substantial heterogeneity in GDM and hypoglycemia meta-analyses remained to be investigated and subgroup analysis was not feasible for all possible factors due to the insufficient number of studies except for baseline BMI of mothers and single- or multiple-strains of probiotics supplementation. Further studies conducted in different races of the population, with larger sample sizes are needed in this regard to validate the health effects of probiotics in pregnant women without GDM.

In conclusion, probiotics supplementation seemed to be able to reduce the risk of GDM incidence and improve glycemic control in pregnant women. Administration before 20 weeks of pregnancy and using multi-strain probiotics are more probable to be effective and *Lactobacillus* was the most popular species used in studies which discovered a preventive effect. However, due to the heterogeneity among existing evidence and small number of studies, results regarding macrosomia, prematurity, preeclampsia, hypoglycemia, NICU admission, cesarean section, birth weight and weight gain are not statistically significant. Further studies are warranted to address these limitations and to reach more definite conclusion.

## Data availability statement

The original contributions presented in the study are included in the article/Supplementary material, further inquiries can be directed to the corresponding author.

## Author contributions

NS, MH, BL, and H-SE contributed to the design of the study. SF, AP, and H-SE search for the related studies. AP and H-SE extracted and analyzed the data. AP wrote the first draft of the manuscript. All the authors contributed to manuscript revision and approved the submitted version.

## Conflict of interest

The authors declare that the research was conducted in the absence of any commercial or financial relationships that could be construed as a potential conflict of interest.

## Publisher's note

All claims expressed in this article are solely those of the authors and do not necessarily represent those of their affiliated organizations, or those of the publisher, the editors and the reviewers. Any product that may be evaluated in this article, or claim that may be made by its manufacturer, is not guaranteed or endorsed by the publisher.
