# Supplementary figures and images for: Preventive effect of probiotics supplementation on occurrence of gestational diabetes mellitus: A systematic review and meta-analysis of randomized controlled trials
Source: Front Med (Lausanne). 2022 Dec 1;9:1031915. doi: 10.3389/fmed.2022.1031915 (PMC9751955; doi:10.3389/fmed.2022.1031915)

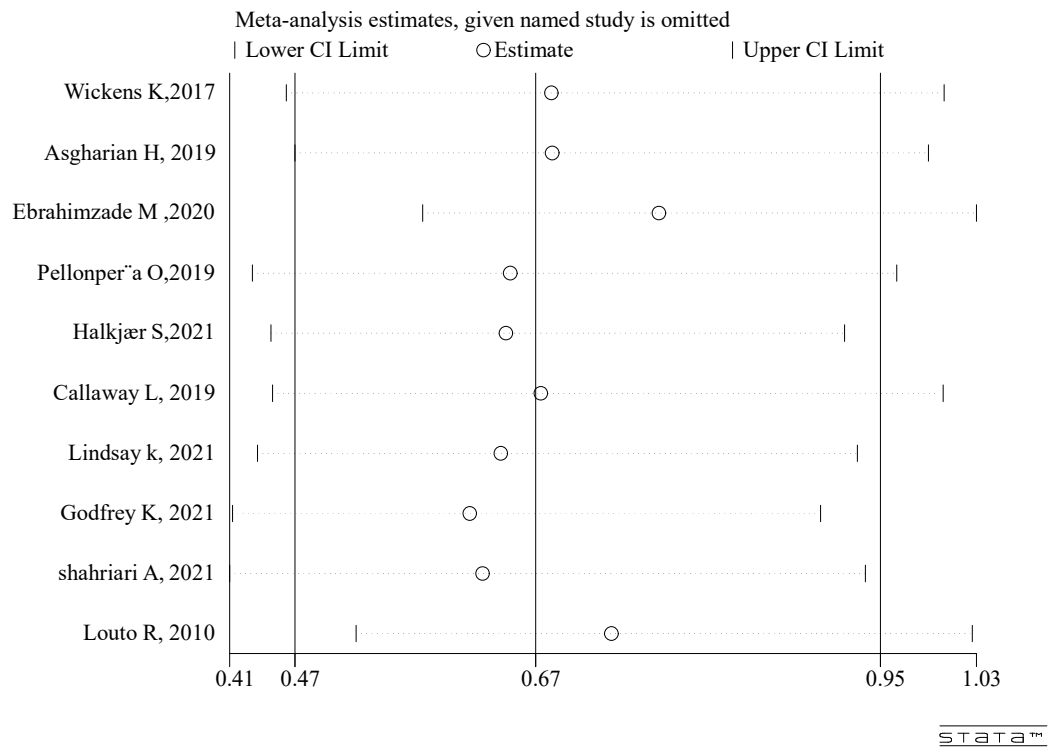

Supplement: Supplementary file 1 [file Data_Sheet_1.PDF]

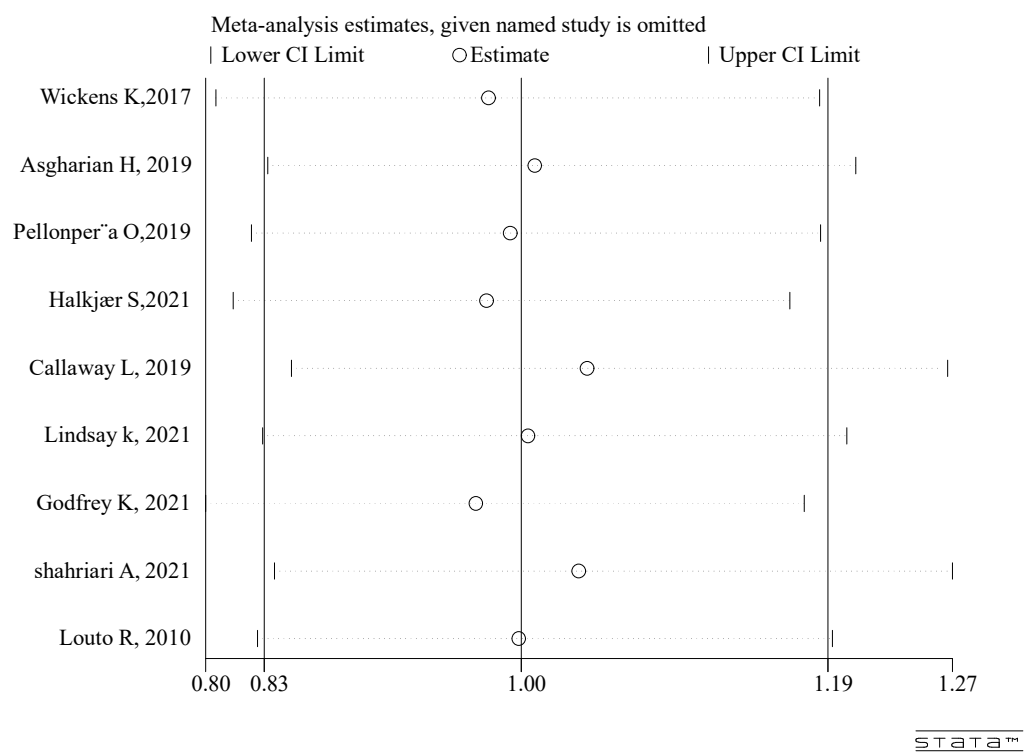

Supplement: Supplementary file 2 [file Data_Sheet_2.PDF]

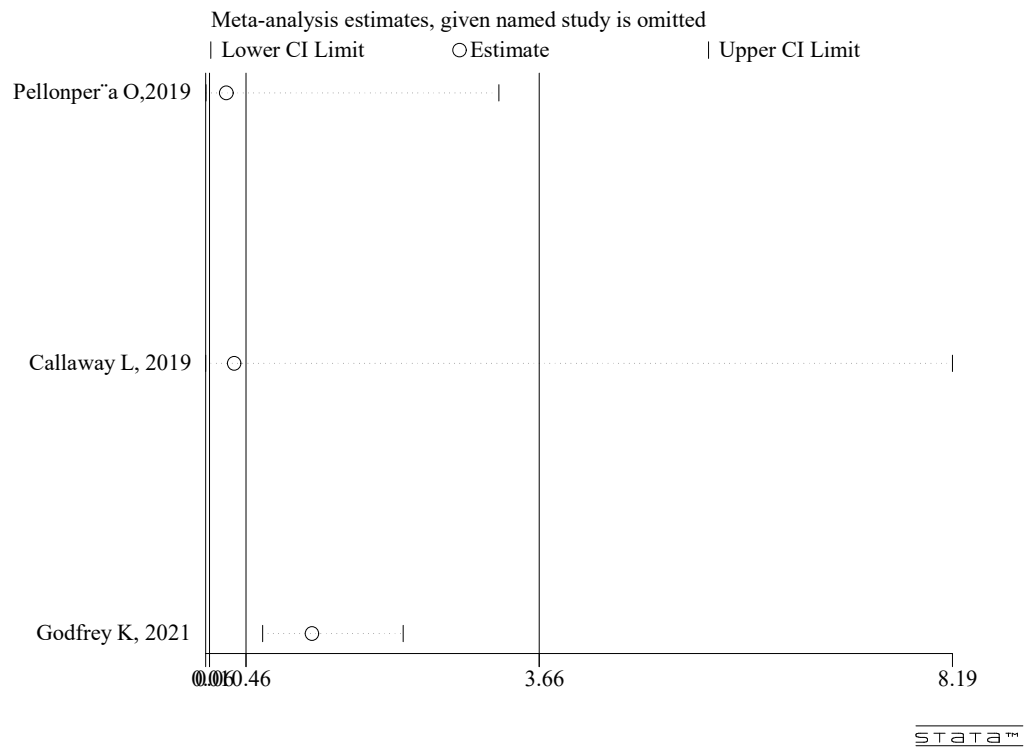

Supplement: Supplementary file 3 [file Data_Sheet_3.PDF]

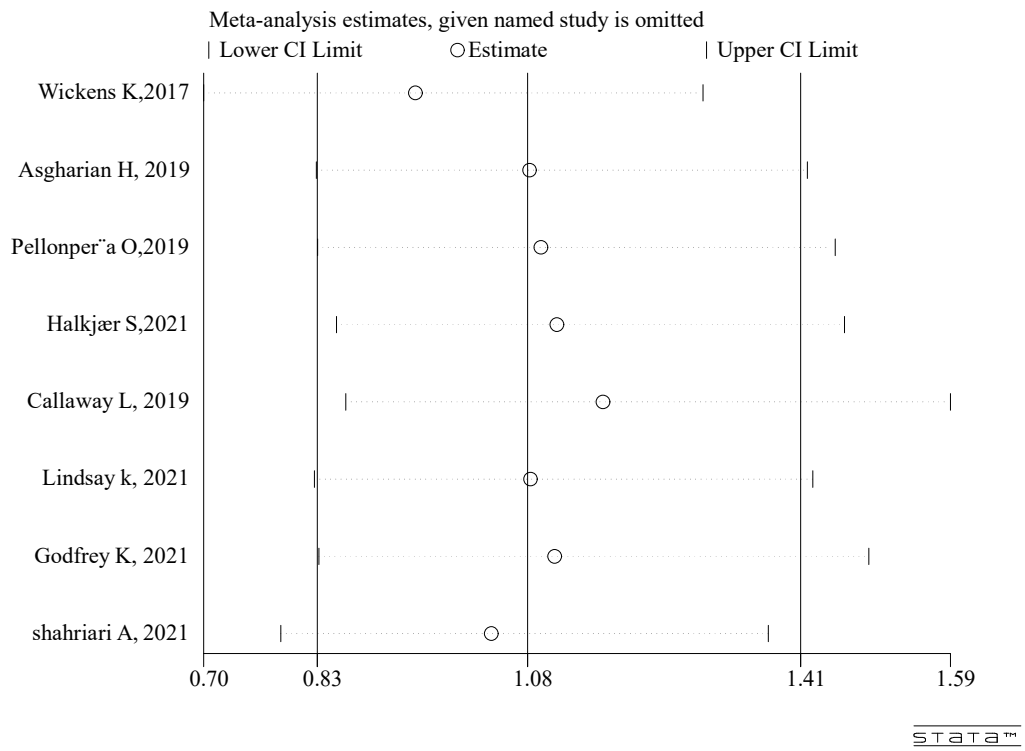

Supplement: Supplementary file 4 [file Data_Sheet_4.PDF]

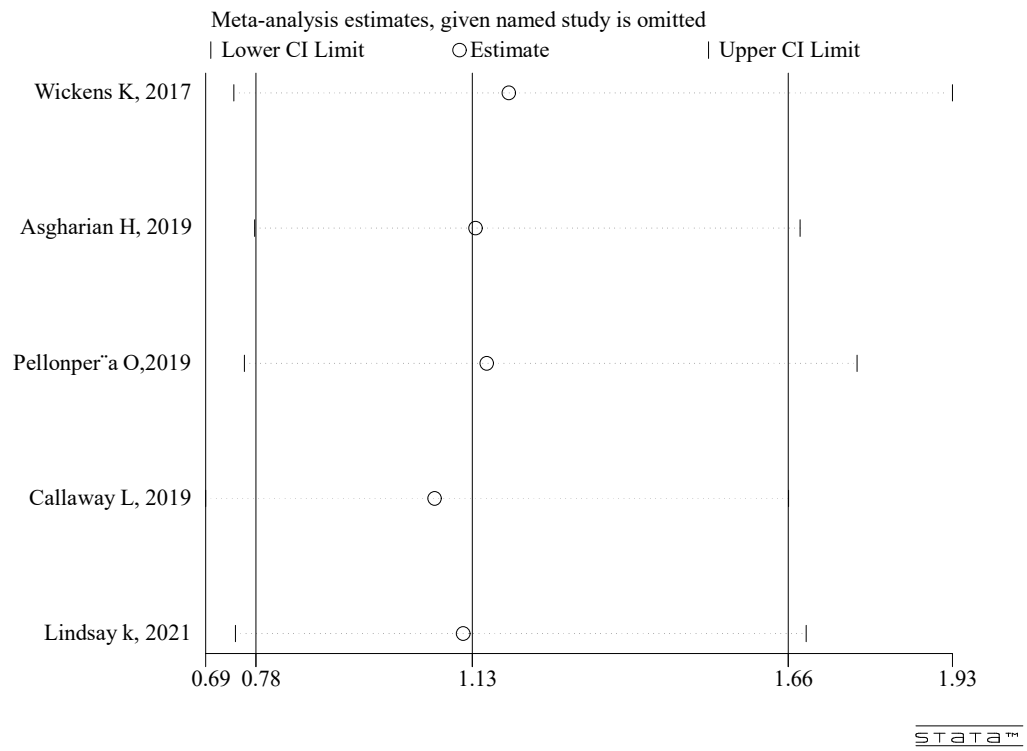

Supplement: Supplementary file 5 [file Data_Sheet_5.PDF]

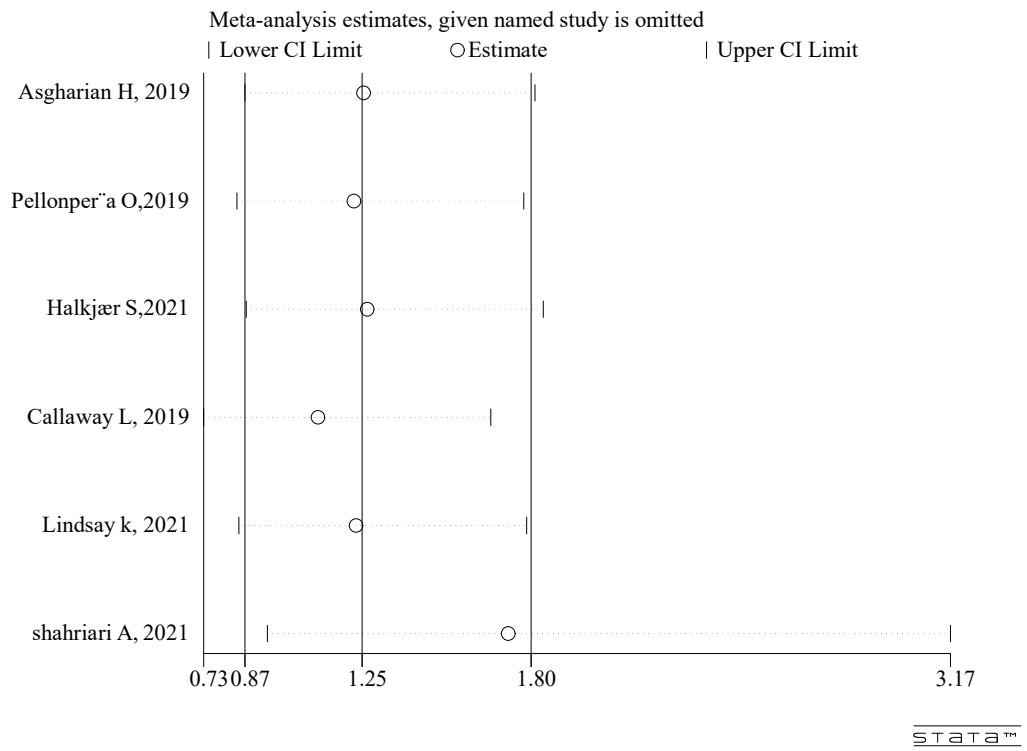

Supplement: Supplementary file 6 [file Data_Sheet_6.PDF]

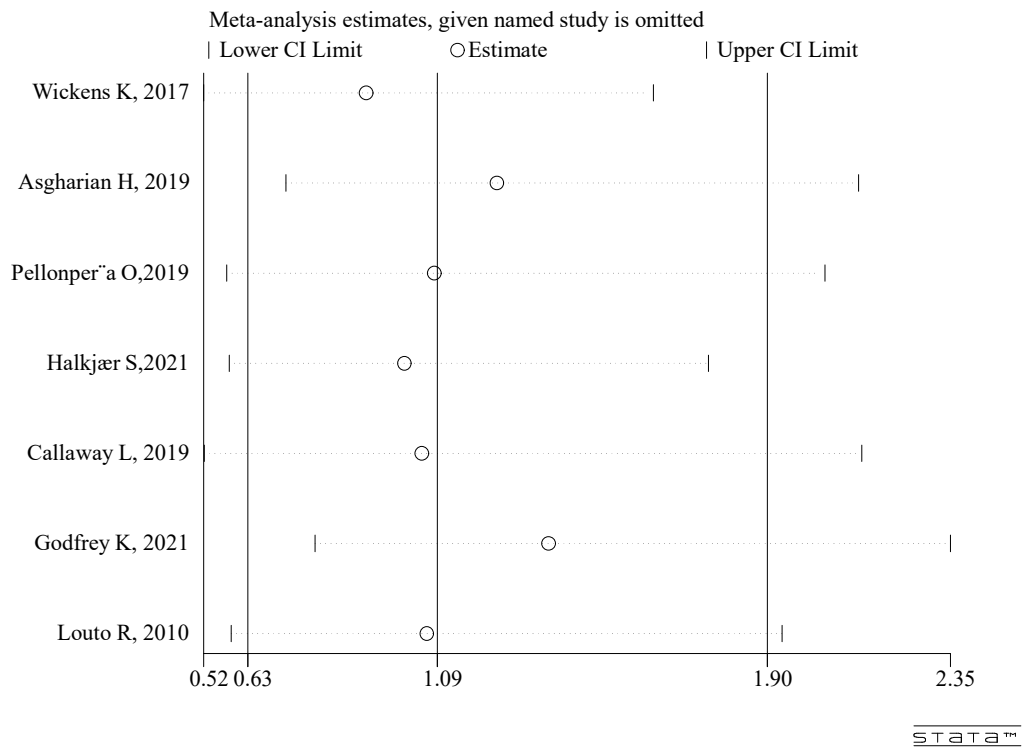

Supplement: Supplementary file 7 [file Data_Sheet_7.PDF]
